# Impact of the COVID-19 pandemic on testing services for HIV, viral hepatitis and sexually transmitted infections in the WHO European Region, March to August 2020

**DOI:** 10.2807/1560-7917.ES.2020.25.47.2001943

**Published:** 2020-11-26

**Authors:** Daniel Simões, Annemarie Rinder Stengaard, Lauren Combs, Dorthe Raben, The EuroTEST COVID-19 impact assessment consortium of partners, The EuroTEST COVID-19 impact assessment consortium of partners, The EuroTEST COVID-19 impact assessment consortium of partners, Anastasia Pharris, Andrew Winter, Ann K Sullivan, Ann-Isabelle von Lingen, Antons Mozalevskis, Cary James, Casper Rokx, Cristina Agusti, Daria Alexeeva, Elena Vovc, Erika Duffel, Giorgi Kuchukhidze, Jordi Casabona, Jürgen K Rockstroh, Justyna D Kowalska, Kristi Rüütel, Lara Tavoschi, Laura Fernandez-Lopez, Magnus Unemo, Maria Buti, Michael Krone, Nicole Seguy, Otilia Mardh, Soudeh Ehsani, Teymur Noori, Valerie Delpech

**Affiliations:** 1 EPIUnit–Instituto de Saúde Pública, Universidade do Porto, Porto, Portugal; 2 Grupo de Ativistas em Tratamentos (GAT), Lisboa, Portugal; 3 Centre of Excellence for Health, Immunity and Infections (CHIP), Rigshospitalet, University of Copenhagen, Copenhagen, Denmark.; 4 The members of the EuroTEST consortium of partners are listed at the end of the article

**Keywords:** Testing, COVID-19, HIV, hepatitis B infection, hepatitis C infection, sexually transmitted infections

## Abstract

We present preliminary results of a coronavirus disease (COVID-19) impact assessment on testing for HIV, viral hepatitis and sexually transmitted infections in the WHO European Region. We analyse 98 responses from secondary care (n = 36), community testing sites (n = 52) and national level (n = 10). Compared to pre-COVID-19, 95% of respondents report decreased testing volumes during March–May and 58% during June–August 2020. Reasons for decreases and mitigation measures were analysed.

The coronavirus disease (COVID-19) continues to challenge healthcare systems across the World Health Organization (WHO) European Region [1-4]. As World AIDS Day 2020 approaches, it serves as a reminder about the importance to maintain a strong HIV response at all levels. Further, as framed in the Sustainable Development Goals (SDGs) [5] and recent European and global guidance [6-8], it is critical to approach testing for HIV, hepatitis B (HBV), hepatitis C (HCV), and sexually transmitted infections (STIs), namely chlamydia, syphilis and gonorrhoea, in an integrated and synergistic manner.

## Challenges for HIV, hepatitis B, hepatitis C and sexually transmitted infections control

Early diagnosis and linkage to care for these infections are key challenges in the WHO European Region, where an estimated one in five people living with HIV do not know their infection status [9], and half are diagnosed at a late stage [9-12]. In the European Union/European Economic Area (EU/EEA), only an estimated 20.3% (2.4–71.8%) of persons with HBV and 26.8% (4.1–96.8%) of those with HCV are aware of their infection [13,14].

The COVID-19 pandemic has required rapid adaptation of health systems and public health measures, at a scale never previously witnessed. Here we report preliminary results of an online survey assessing the impact of COVID-19 on testing for HIV, HBV, HCV and STIs in the 53 countries of the WHO European Region.

The survey (Supplementary Table S1) was developed by a consortium of partners [15] and distributed through the consortium member’s respective networks between 14 October and 13 November 2020 to a wide range of actors involved in the provision of testing services in the WHO European Region (laboratories, primary care units, secondary level care clinics, community sites and national level public health institutions or ministries of health). Key questions comprised the quantitative impact on testing volume (asked in broad categories of percentage decrease/increase), main reasons for the observed impact, measures put in place to mitigate it and areas where guidance or support were needed.

## Magnitude of decline in testing

A total of 98 survey responses, received as at 10 November 2020, were included in this preliminary analysis. They include 52 community and non-governmental organisations (CBOs and NGOs) providing testing for at least one of these infections, 36 secondary level/specialist healthcare (SHC) providers, and 10 national level public health institutes or ministries of health (NLPHM) from 34 countries in the WHO European Region (23 EU/EEA and 11 non-EU/EEA) (Supplementary Table S2). Survey responses from laboratories (n = 5) and primary healthcare facilities (n = 5) were not included in this analysis, because of the low numbers of respondents.

**Figure 1:**
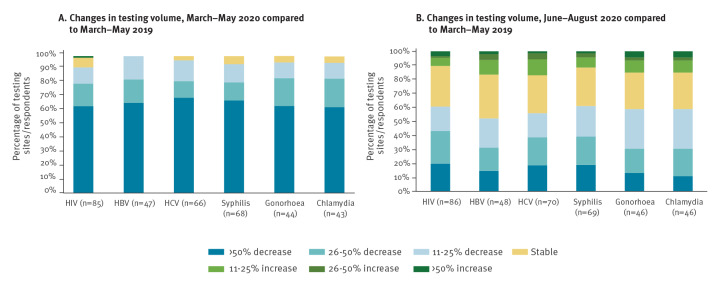
Changes in testing volume for HIV, HBV, HCV and STIs^a^ by infection and category of change in (A) March–May 2020 and (B) June–August, compared with March–May 2019 in 34 WHO European Region countries (n = 98 respondents)

HBV: hepatitis B virus; HCV: hepatitis C virus; STI: sexually transmitted infections; WHO: World Health Organization.

^a^ Chlamydia, syphilis and gonorrhoea.

Across infections, almost all (95%; 92/98) respondents reported testing decreases from March to May 2020 as compared with the same period in 2019, including 64% (63/98) who reported severe disruptions to testing provision (> 50% decline in testing volume) (Figure 1). Between June and August 2020, 58% (57/98) still reported testing volume decreases, however, the degree of disruption was less severe with 20% (20/98) reporting > 50% declines in testing and 14% (14/98) even reporting increases in testing volume (Figure 1).

These findings were consistent across all infections surveyed, with between nine of 86 HIV testing providers and eight of 48 HBV testing providers reporting increases in testing volume from June to August 2020 (Figure 1).

**Figure 2:**
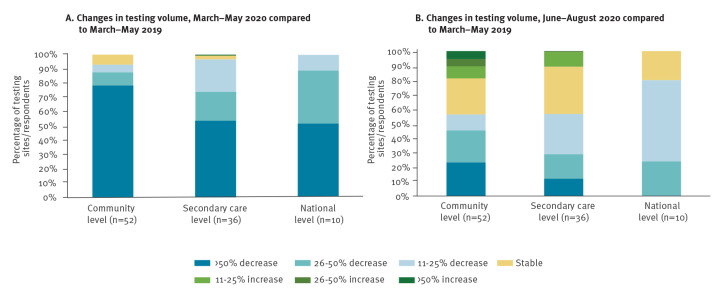
Changes in testing volume for HIV, HBV, HCV and STIs^a^, by setting and category of change in (A) March–May 2020 and (B) June–August 2020, compared with March–May 2019, in 34 WHO European Region countries (n = 98 respondents)

HBV: hepatitis B virus; HCV: hepatitis C virus; STIs: sexually transmitted infections; WHO: World Health Organization.

^a^ Chlamydia, syphilis and gonorrhoea.

Although not directly comparable, severe disruption in testing services (≥ 50% decrease) were reported by all settings for the period March to May 2020 compared with March to May 2019 (Figure 2). Assessing the average change across all infections, 41/52 of community-based testing sites and 19/36 of secondary level care sites reported severe testing disruptions. For the period June-August 2020 (compared with the same pre-COVID baseline), fewer sites reported severe disruptions: 12/52 of community sites and 4/36 of secondary level sites. National level data (based on 10 responses) suggested a >50% decrease in five countries in the early period and smaller decreases in the later period (Figure 2).

Reported estimations of positivity tended to decrease or remain stable during March to May 2020 and remained stable or increased slightly from June to August, in comparison with a March 2019 baseline (data not shown).

Reasons for the observed decreases in testing volume (Table 1) included (in order of frequency reported): testing site closure during lockdown, reduced staff, reduced attendance and fewer appointments scheduled, fewer serological samples drawn, laboratories overburdened and fewer referrals to the facility.

**Table 1:** Reported reasons^a^ for observed declines in testing volumes for HIV, HBV, HCV and STIs^b^ and measures implemented to restore testing provision^c^, by setting, in 34 WHO European Region countries, March–August 2020 (n = 98 respondents)

	Community level sites (n = 52)	Secondary level care sites (n = 36)	National level (n = 10)
	n	n	n
**Reasons for observed declines in testing volume**			
Testing site(s) closed during lockdown	36	21	6
Staff re-allocated to support COVID-19 response	8	15	5
Reduced staff in testing site	31	11	NA
Fewer appointments scheduled/reduced attendance	36	28	8
Fewer serological samples drawn and sent to the laboratory/fewer referrals to blood draw/testing	10	24	NA
No ‘drop-in’ service (only testing by appointment)	26	15	NA
Fewer referrals to site	13	18	NA
Changes in financing system	10	4	0
Stock-out of test kits, tubes, reagents or consumables	3	5	1
Triaging of patients^d^	16	12	2
Moved to telemedicine/remote consultations	20	14	NA
Laboratories overburdened	NA	NA	4
Other	2	3	0
**Measures implemented to restore testing provision**			
Remote counselling appointments	35	25	0
Home-based sampling	8	6	2
HIV self-testing (on-site or by referral)	23	4	1
Triaging of patients^d^	16	13	0
No ‘drop-in’ service (only testing by appointment)	25	13	NA
Referral to other testing sites	19	6	NA
Staff reinforcement	5	8	0
Funding reallocations	13	1	NA
Equipment acquisition	5	0	NA
Expanded outreach testing	11	4	NA
Testing campaigns	19	4	NA
Revised diagnosis algorithm	NA	NA	0
Community based testing	NA	NA	0
Lay provider testing	NA	NA	1

COVID-19: coronavirus disease; HBV: hepatitis B virus; HCV: hepatitis C virus; NA: not asked to this group; STIs: sexually transmitted infections; WHO: World Health Organization.

^a^ For each reported reason, respondents were asked to indicate if they perceived the impact level as major, medium or minor, responses here include major and medium.

^b^ Chlamydia, syphilis and gonorrhoea.

^c^ Each respondent could indicate more than one reason or new measure, hence the totals per column are higher than the number of respondents.

^d^ Stricter criteria for who is being offered testing.

## Measures to mitigate decline in testing

Respondents reported a range of new measures implemented to mitigate the impact on testing. Remote appointments were overall the most frequently reported (25/36 of SHC and 35/52 of CBO and NGO sites). Both settings also introduced stricter criteria for test offer (13/36 SHC sites and 16/52 CBO and NGO), appointment-only testing (13/36 SHC sites, 25/52 CBO and NGO) and referral to other testing sites (6/36% SHC, 19/52 CBO and NGO).

In SHC sites, other new measures included staff reinforcements (8/36) and home-based sampling (6/36). In CBOs and NGOs, self-testing (23/52), testing campaigns (19/52) funding reallocations (13/52) and expanded outreach (22/52) were reported.

At the national level, regarding changes in testing modalities available, home-based sampling (2/10), HIV self-testing (1/10) and lay provider testing (1/10) were reported (Table 1).

## Needs for guidance or support

Regarding guidance or support needs (Table 2), the most frequently reported ones were additional human resources (16/36 for SHC and 23/52 for CBO and NGO), and increased financial support (35/52 of CBO and NGO; 13/36 of SHC). One third (16/52) of CBO and NGO listed regulatory changes as important. Regulatory issues mentioned were regulation of self-testing and community based/lay provider testing, as well as adapted guidance for CBO and NGO to continue to provide services during lockdown, removing or revising prohibitive legislations or regulations associated with specific practices or key populations (such as abortion, sex work or drug use). At the national level, four respondents stated that no particular support was needed while two mentioned programmatic guidance as important.

**Table 2:** Guidance or support considered important to reduce the impact of COVID-19 on testing for HIV, HBV, HCV and STIs^a^, by setting, in 34 WHO European Region countries, March–August 2020 (n = 98 respondents)

	Community level sites (n = 52)	Secondary level care sites (n = 36)	National level (n = 10)
Additional human resources	23	16	1
Increased financial support	35	13	1
Regulatory changes	16	2	0
Programmatic guidance	4	1	2
Technical guidance	6	3	1
Technical support on specific issue	5	4	1
Procurement/supply chain related support	6	4	1
Other	4	2	0
None	7	5	4

COVID-19: coronavirus disease; HBV hepatitis B virus; HCV hepatitis C virus; STIs: sexually transmitted infections; WHO: World Health Organization.

^a^ Chlamydia, syphilis and gonorrhoea.

## Discussion and conclusions

The COVID-19 pandemic has had considerable impact on testing for HIV, viral hepatitis and STIs in the WHO European Region. Our preliminary results show that 95% of respondents from 34 countries reported testing less than half the expected number of people during the first months of the COVID-19 pandemic between March and May 2020. This continued, although to a lesser degree, between June and August 2020, when measures were less strict in most countries. These findings are indicating an important decline in testing volumes; however, it should be kept in mind that they represent broad categories of reported change and are based on best estimates rather than accurate data for some respondents.

For chronic infections such as HIV and viral hepatitis, delayed diagnosis and treatment may result in further long-term consequences including sequelae for individual patients [16,17], and even a stalling of progresses achieved so far in controlling these infections in the WHO European Region.

Given the high reported impact on testing in community services, also documented in other reports [18-20], where efforts focus on key populations, our findings suggest that these vulnerable populations may have experienced further reduced access to testing and other essential services than before the pandemic.

With reported cases of COVID-19 being on the rise in the Region in the autumn of 2020 [1,2] and movement restrictions being re-imposed in many countries [21,22], support and guidance are paramount to minimise testing interruptions and ensure the long term sustainability of existing HIV, hepatitis and STIs testing services. Guidance should include contingency planning and development of COVID-secure testing services in all settings.

The survey respondents report several new measures implemented because of the pandemic. Although these do not reflect an effort to recover testing operations, they aim to provide minimum services or adapt testing capacity. Complementary testing modalities for existing testing strategies such as self-testing (for HIV) and self-sampling (for HIV, hepatitis and STIs), had not been widely implemented before the pandemic [12]. They constitute important options to diversify and optimise access to testing that should be regulated and made available as part of policy and practice at a national level.

The EU/EEA and other Regional partners can play an instrumental role in accelerating stakeholder involvement, regulatory development and integration processes, including fostering cross-border cooperation and supporting exchange and implementation of best practices, as done through several European projects focusing on HIV, viral hepatitis and STI and bringing together different actors [6,23-26].

The COVID-19 response in several countries of the WHO European Region has shown capacity in rapidly responding to health threats, which has translated into the rapid scale-up of COVID-19 testing [27], new/innovative initiatives to improve contact tracing for this infection [28] and implementation of adapted HIV, viral hepatitis and STI testing and linkage to care solutions [29,30]. This has shown that it is possible to mobilise resources and adapt policies in a short time, despite limited scientific evidence. These learnings can be transferred to the response planning of HIV and other infectious diseases, for which there is a larger evidence base and agreed SDGs.

As the COVID-19 pandemic - and future emerging infectious diseases - will likely remain a priority in the Region, it is important to ensure that the response to other infectious diseases is not compromised. Investing in integrated testing responses through dialogue of those involved at all levels, including for HIV, viral hepatitis and STIs, can speed up recovery of testing provision in the Region, particularly in the case of key populations, which often are at a higher risk of acquiring more than one infectious disease.

## The EuroTEST COVID-19 impact assessment consortium of partners

Anastasia Pharris, ECDC, Stockholm, Sweden; Andrew Winter, International Union against Sexually Transmitted Infections (IUSTI) Europe, Glasgow, United Kingdom; Ann K Sullivan, Chelsea and Westminster Hospital NHS Foundation Trust, London, United Kingdom; Ann-Isabelle von Lingen, European AIDS Treatment Group (EATG), Brussels, Belgium; Antons Mozalevskis, WHO Regional Office for Europe, Copenhagen, Denmark; Cary James, World Hepatitis Alliance (WHA), London, United Kingdom; Casper Rokx, Erasmus University Medical Center, Rotterdam, the Netherlands; Cristina Agusti, Centre d’Estudis Epidemiològics sobre les ITS i Sida de Catalunya (CEEISCAT) - Centro de Investigación Biomédica en Red de Epidemiologia y Salud Pública (CIBERESP), Barcelona, Spain; Daria Alexeeva, AFEW International, Amsterdam, the Netherlands; Elena Vovc, WHO Regional Office for Europe, Copenhagen, Denmark; Erika Duffel, ECDC, Stockholm, Sweden; Giorgi Kuchukhidze, WHO Regional Office for Europe, Copenhagen, Denmark; Jordi Casabona, Centre for Epidemiological Studies on Sexually Transmitted Infections and HIV/AIDS of Catalonia (CEEISCAT), Catalan Health Department, Centros de Investigación Biomédica en Red Epidemiología y Salud Pública (CIBERESP), Barcelona, Spain; Jürgen K Rockstroh, Department of Medicine I, Univeristy Hospital Bonn, Bonn, Germany; Justyna D Kowalska, Medical University of Warsaw, Warsaw, Poland; Kristi Rüütel, National Institute for Health Development, Tallinn, Estonia; Lara Tavoschi, University of Pisa, Pisa, Italy; Laura Fernandez-Lopez, Centre for Epidemiological Studies on Sexually Transmitted Infections and HIV/AIDS of Catalonia (CEEISCAT), Catalan Health Department, Centros de Investigación Biomédica en Red Epidemiología y Salud Pública (CIBERESP), Barcelona, Spain; Magnus Unemo, WHO Collaborating Centre for STIs, Örebro University, Örebro, Sweden; Maria Buti, European Association for the Study of the Liver (EASL), Barcelona, Spain; Michael Krone, Aids Action Europe (AAE), Berlin, Germany; Nicole Seguy, WHO Regional Office for Europe, Copenhagen, Denmark; Otilia Mardh, ECDC, Stockholm, Sweden; Soudeh Ehsani, WHO Regional Office for Europe, Copenhagen, Denmark; Teymur Noori, ECDC, Stockholm, Sweden; Valerie Delpech, Public Health England, London, United Kingdom.

## Supplementary Data

611000 Supplementary Material
