# Supplementary material for: Impact of the COVID-19 pandemic on testing services for HIV, viral hepatitis and sexually transmitted infections in the WHO European Region, March to August 2020
Source: Euro Surveill. 2020 Nov 26;25(47):2001943. doi: 10.2807/1560-7917.ES.2020.25.47.2001943 (PMC7693166; doi:10.2807/1560-7917.ES.2020.25.47.2001943)
Supplement: Supplementary Material [file 20-01943_STENGAARD_Supplementary_material.pdf]

This supplementary material is hosted by *Eurosurveillance* as supporting information alongside the article 'Impact of the COVID-19 pandemic on testing services for HIV, viral hepatitis and sexually transmitted infections in the WHO European Region, March to August 2020', on behalf of the authors, who remain responsible for the accuracy and appropriateness of the content. The same standards for ethics, copyright, attributions and permissions as for the article apply. Supplements are not edited by *Eurosurveillance* and the journal is not responsible for the maintenance of any links or email addresses provided therein.

Supplementary Table S1. COVID-19 impact assessment on testing for HIV, viral hepatitis and STIs in the WHO European Region: Survey questions

**BACKGROUND**	
**Site identification and description, including infections tested for:**	
1. Which country are you based in/do you primarily work in?	*Drop down menu*
2. Please provide the name of your organisation:<br><br>*Site names are requested exclusively to avoid duplication of responses and will remain accessible solely to the research team and not be published in any identifiable format.*	*Free text*
3. What type of facility/institution are you responding on behalf of? (single choice)	a. Laboratory<br>b. Secondary level/specialist care<br>c. Primary health care (general practitioners, primary care teams)<br>d. Community-based testing site (e.g. NGO, CBO, private/public VCT)<br>e. National Public Health Institute or Ministry of Health
a. Please indicate the type of laboratory:	a. National reference laboratory<br>b. Regional laboratory<br>c. Local/city-level laboratory<br>d. Hospital laboratory<br>e. Other (please specify)
b. Please describe your laboratory according to the following COVID-19 related categories	a. Laboratory performing COVID-19 PCR diagnostics<br>b. Laboratory performing COVID-19 serological/antibody testing<br>c. POC and/or near POC testing<br>d. Other<br>e. Do not perform COVID-19 testing
c. Please indicate the specialty/specialties that your response represents and if the facility is part of a larger hospital (tick all that apply)	a. STI clinic/unit<br>b. HIV clinic/unit<br>c. Hepatology unit<br>d. TB clinic/unit<br>e. Gynaecology clinic/unit<br>f. Multiple specialties/integrated clinic/unit<br>g. Other specialty (please specify)
d. Please indicate the type of site (NGO, CBO, public VCT etc.) and number of testing sites that your response represents	
e. Please indicate the type of primary health care site your response represents:	
4. Please provide your email address:<br>*Please note: We will use and store your email address exclusively to contact you if we need clarification regarding your survey response or to identify duplicate responses. We will not share it with any other organisation or use it for any other purpose than stated here.*	*By providing my email address, I allow EuroTEST to contact me with validation questions related to this survey.*

5. Please indicate which infections your survey response will cover (i.e. which infections your service provides testing for, or, if you work in a national level institution, for which you are responsible)	a. HIV<br>b. HBV<br>c. HCV<br>d. Syphilis<br>e. Chlamydia<br>f. Gonorrhoea
6. *(Not for NFP)* Please provide the average number of people tested in an average month in 2019 (i.e. pre-COVID-19). Please provide your best estimate/range and include all types of screening/diagnostic test types per infection.	*Additional free text response option for each disease marked in question 5*
a. Please provide the average number of people tested for HIV in an average month in 2019 (i.e. pre-COVID-19).	
b. Please provide the average number of people tested for HBV in an average month in 2019 (i.e. pre-COVID-19).	
c. Please provide the average number of people tested for HCV in an average month in 2019 (i.e. pre-COVID-19).	
d. Please provide the average number of people tested for syphilis in an average month in 2019 (i.e. pre-COVID-19).	
e. Please provide the average number of people tested for chlamydia in an average month in 2019 (i.e. pre-COVID-19).	
f. Please provide the average number of people tested for gonorrhoea in an average month in 2019 (i.e. pre-COVID-19).	
7. *(Not for NFP)* Does your facility provide other forms of services (provided before the COVID-19 pandemic) (tick all that apply)?	a. Needle and syringe exchange<br>b. Opioid substitution therapy<br>c. HIV self-testing (offering or referring)<br>d. Home-based sampling<br>e. Partner notification<br>f. Referral/support in linkage to care or confirmatory testing<br>g. PrEP (initiation, provision or monitoring)<br>h. Mental health support<br>i. Social support<br>j. Remote consultations (phone or email)<br>k. Other (please provide)
8. *Only for non-EU/EEA countries (including NFP)* Did your country impose movement restrictions that could affect people's access to health services (e.g. mandatory 'stay-at-home' orders, 'stay-at-home' recommendations for risk groups, closure of public spaces etc.)	a. Yes<br>b. No
a. If yes, please indicate which type of restrictions (tick all that apply)	a. Mandatory 'stay-at-home' orders for the general population<br>b. Recommended/optional 'stay-at-home' orders for the general population<br>c. Optional 'stay-at-home' recommendations for risk groups or vulnerable populations<br>d. Closure of public spaces (for example restaurants, entertainment venues, non-essential shops, partial or full closure of public transport etc.)<br>e. Other (please specify)
Please provide any additional comments or explanations	*Free text field*

**Impact of COVID-19 on HIV, viral hepatitis and STI testing**								
**Quantitative impact on HIV, viral hepatitis and STI testing (diagnostic or screening), March-May 2020**<br>In comparison with the same three months last year (1 March to 31 May 2019), please indicate the relative change in the number of people tested (across all screening/diagnostic test types per infection) in your site/facility/country in the period 1 March to 31 May 2020. (If accurate data are not available to calculate the change, then please provide your best estimate/judgment):								
Infection	Decreased by			Stable	Increased by			No data
	>50%	26-50%	11-25%	0-10% (+/-)	11-25%	26-50%	>50%	
HIV								
HBV								
HCV								
Syphilis								
Gonorrhoea								
Chlamydia								
Please provide any additional comments or explanations you find relevant				*Free text field*				
**Quantitative impact on HIV, viral hepatitis and STI testing (diagnostic or screening), June-August 2020**<br>in the number of people tested (across all screening/diagnostic test types per infection) in your site/facility/country in the period 1 June to 31 August 2020<br>If accurate data are not available to calculate the change, then please provide your best estimate/judgment:								
Infection	Decreased by			Stable	Increased by			No data
	>50%	26-50%	11-25%	0-10% (+/-)	11-25%	26-50%	>50%	
HIV								
HBV								
HCV								
Syphilis								
Gonorrhoea								

Chlamydia								
Please provide any additional comments or explanations you find relevant				*Free text field*				

(FOR ALL SETTINGS EXCEPT LABORATORIES AND NATIONAL LEVEL RESPONDENTS)<br>**Reasons for any observed declines in HIV/HCV/HBV/STI testing volume**<br>If you observed a decrease in testing volume for any of the infections indicated above, please provide the reasons and indicate if the impact-level was Major, Medium, Minor, Not applicable, Do not know, or No decrease.<br>Please also elaborate in the comment box below if the reasons differed across the different infections.						
	Major	Medium	Minor	Not applicable	Do not know	No decrease
Facility/site closed during lockdown						
Staff re-allocated to support the COVID-19 response						
Reduced staff in facility/site (due to illness, working remotely, being in lockdown, reduced opening hours etc.)						
Fewer appointments scheduled/reduced attendance						
Fewer serological samples drawn and sent to the laboratory/fewer referrals to blood draw/testing						
No 'drop-in' service (only testing by appointment)						
Fewer referrals to your facility (for specialist clinics/units and laboratories)						
Changes in financing system						
Stock-out of test kits						
Triaging of patients (stricter criteria for who is being offered testing)						
Moved to telemedicine (remote consultations, phone or online)						
Other						
If Yes, to "Staff re-allocated to support the COVID-19 response," can you estimate the % of human resources usually supporting HIV/HBV/HCV/STI testing that was reallocated to COVID-19?						
Please provide any additional comments or explanations you find relevant to explain the decrease in testing						

(FOR NATIONAL LEVEL RESPONDENTS ONLY)<br>**Reasons for any observed declines in HIV/HCV/HBV/STI testing volume**<br>If you observed a decrease in testing volume for any of the infections indicated above, please provide the reasons and indicate if the impact-level was Major, Medium, Minor, Not applicable, Do not know, or No decrease.<br><br>Please also elaborate in the comment box below if the reasons differed across the different infections.						
	Major	Medium	Minor	Not applicable	Do not know	No decrease
Many testing sites were closed during lockdown						

Many sites had reduced opening hours						
Staff re-allocated to support the COVID-19 response						
Fewer appointments scheduled/reduced attendance						
Laboratories overburdened						
Stock-out of test kits						
Revised/stricter criteria for who is being offered testing						
Changes in the financing system						
Other						
Please provide any additional comments or explanations you find relevant to explain the decrease in testing						
If yes, can you estimate the % of human resources usually supporting HIV/HBV/HCV/STI testing that was reallocated to COVID-19?						

(FOR LABORATORIES ONLY)

Reasons for any observed declines in HIV/HCV/HBV/STI testing volume

If you observed a decrease in testing volume for any of the infections indicated above, please provide the reasons and indicate if the impact-level was Major, Medium, Minor, Not applicable, Do not know, or No decrease.

Please also elaborate in the comment box below if the reasons differed across the different infections.

	Major	Medium	Minor	Not applicable	Do not know	No decrease
Laboratory closed during lockdown						
Staff normally supporting HIV/HCV/HBV/STI testing was re-allocated to support COVID-19						
Equipment normally used for serologic HIV/HCV/HBV/syphilis testing was re-allocated to support COVID-19 testing						
Equipment normally used for PCR-based HIV/HCV/HBV/STI testing was re-allocated to support COVID-19 testing						
Funding normally used for HIV/HCV/HBV/STI testing was reallocated to support COVID-19						
Reduced opening hours etc./no open 'drop-in' times						
Fewer appointments scheduled/fewer referrals to your lab						
Fewer samples sent to your lab						
Stock-out of sampling tubes, reagents or consumables for HIV/HBV/HCV/STI testing						
Triaging of patients (stricter criteria for who is being offered testing)						
Other						
Please provide any additional comments or explanations you find relevant to explain the decrease in testing						

(FOR NATIONAL LEVEL RESPONDENTS ONLY)

Changes to national level HIV, viral hepatitis and STI testing policies and practices generated as a result of the COVID-19 pandemic

Please indicate if any national level policies or practice were changed to mitigate the impact of the COVID-19 pandemic on testing for HIV, viral hepatitis and STIs in your country (please check all that apply)?

	Unchanged	Newly implemented	Changed	Not implemented but planned within the short term (6-months)	Not implemented but planned within the medium/long term (1 year+)	Please briefly describe the change (implemented, changed or planned) *Free text*	No information
Recommendations on who to test and how frequently to test (for example stricter criteria for who is being offered testing)							
Revised national diagnostic algorithm (e.g. simplifying the process for confirmatory diagnosis/ reducing the need for laboratory confirmation, introduction of new RNA/antigen tests etc.)							
HIV self-testing* *(only to those responsible for HIV at national level)*							
Home-based sampling*							
Community-based testing							
Lay provider testing							
Funding allocation							
Other (please specify)							

(FOR NATIONAL LEVEL RESPONDENTS ONLY)

Changes to other national level HIV, viral hepatitis and STI policies and practices generated as a result of the COVID-19 pandemic

4. Please indicate if any national level policies or practices were changed to mitigate the impact of the COVID-19 pandemic on other HIV, viral hepatitis and STIs services in your country (please check all that apply)?

	Unchanged	Newly implemented	Changed	Not implemented but	Not implemented but	Please briefly describe	No infor

				planned within the short term (6-months)	planned within the medium/long term (1 year+)	the change (implemented, changed or planned) *Free text*	mation
Linkage to care							
Partner notification							
PrEP initiation *(only to those responsible for HIV)*							
PrEP monitoring *(only to those responsible for HIV)*							
Needle and syringe exchange programmes *(only to those responsible for HIV and viral hepatitis)*							
Opioid substitution therapy programmes *(only to those responsible for HIV and viral hepatitis)*							
Surveillance and response monitoring							
Other (please specify)							

(NOT FOR NATIONAL LEVEL RESPONDENTS OR LABORATORIES)<br>**Change in the profile of people accessing testing**	
a. Have you observed any different trends in the profile of people who have been accessing your testing site during the pandemic, in comparison with an average month pre-COVID (e.g. changes by age or sex/gender or in terms of key population groups at higher risk of infection)?	a. Yes (if Yes, please elaborate)<br>b. No<br>c. Do not know

(NOT FOR NATIONAL LEVEL RESPONDENTS OR LABORATORIES)<br>**Positivity rate**	
b. To assess positivity rate, are you able to provide data on monthly positivity of tests performed at your site (i.e. the percentage of tests performed for which the result was positive)?	a. Yes *(if yes, please insert data below)*<br>b. No *(If no, branch into new question below)*
a. Based on your best qualitative judgment, do you consider that the positivity rate has increased, decreased or remained stable in the period from 1 March to 31 May 2020 in comparison with the same period in 2019?	a. Increased<br>b. Decreased<br>c. Remained stable<br>d. Don't know
b. Based on your best qualitative judgment, do you consider that the positivity rate has increased, decreased or remained stable in the period from 1 June to 30 Aug 2020 in comparison with the same period in 2019?	a. Increased<br>b. Decreased<br>c. Remained stable<br>d. Don't know
c. Comments regarding positivity rate	

In the table below, please provide the percentage of tests performed for which the result was positive per infection.<br>(For example, 5% of HIV tests performed were positive in March 2020)<br>Please provide the data in percentages, if no data is available, please type "No data" in the relevant field.						
Month	HIV Positivity	HBV Positivity	HCV Positivity	Syphilis Positivity	Chlamydia Positivity	Gonorrhoea Positivity
Baseline (March 2019)						
March 2020						
April 2020						
May 2020						
June 2020						
July 2020						
August 2020						

(FOR LABORATORIES ONLY)<br>**Changes in testing volume for antenatal care screening, blood donor screening, clinical patient monitoring or and HPV screening**					
If your laboratory conducts antenatal care screening, did the volume of tests decrease in the period 1 March to 31 August 2020?<br>If yes, please indicate your estimated percentage decrease in the table below per infection					
	10-25%	26-50%	>50%	We do not offer these tests	Do not know
HIV					
HBV					
Syphilis					
If your laboratory conducts blood donor screening, did the volume of tests decrease in the period 1 March to 31 August 2020?<br>If yes, please indicate your estimated percentage decrease in the table below per infection					
	10-25%	26-50%	>50%	We do not offer these tests	Do not know
HIV					
HBV					
HCV					
Syphilis					
If your laboratory conducts HIV/HCV/HBV clinical patient monitoring samples (e.g. viral load, liver enzymes, CD4 etc.), did the volume of tests decrease in the period 1 March to 31 August 2020?<br>If yes, please indicate your estimated percentage decrease in the table below per infection					
	10-25%	26-50%	>50%	We do not offer these tests	Do not know
HIV					
HBV					
HCV					
If your laboratory conducts HPV screening, did the volume of tests decrease in the period 1 March to 31 August 2020?			a. Yes<br>b. No<br>c. We do not offer these tests<br>d. Do not know		

If "Yes," please indicate your estimated percentage decrease HPV screening	a. 10-25%<br>b. 26-50%<br>c. >50%
Please elaborate on your response as relevant, e.g. observed impact of COVID-19 on the listed screening programmes	
**Delays in communicating tests results to clinicians or patients/users**	
In the period 1 March to 30 August 2020, were there significant delays in communicating tests results to clinicians or patients/users (ie results took an excess of over two working days or more to be returned)?	a. Yes<br>b. No
If Yes, for which diagnostic tests (excluding blood donor and ANC screening) (Select all that apply)	a. HIV<br>b. HBV<br>c. HCV<br>d. Syphilis<br>e. Chlamydia<br>f. Gonorrhoea
If Yes, for which patient monitoring tests (viral loads, liver enzymes, CD4 etc.) (Select all that apply)	a. HIV<br>b. HBV<br>c. HCV
Were there changes in the national guidelines for HIV/HCV/HBV/STI diagnosis (diagnostic algorithm) or patient monitoring since the beginning of the COVID-19 pandemic?	a. Yes<br>b. No
If Yes, please specify	

(Only for community and PHC respondents)<br>**Impact on linkage to care**			
For people testing/screening positive for HIV, viral hepatitis or and STI in your facility/site, did you experience problems in ensuring linkage to relevant health care services?	a. Yes<br>b. No<br>c. Do not know		
If yes, please indicate the main challenges *(tick all that apply)*:	a. There were difficulties in contacting the specialist care unit(s) normally referred to<br>b. There were delays in scheduling consultations<br>c. No referral to specialists was possible, except for emergency situations<br>d. No elective procedures/ investigations were possible, except emergency situations<br>e. Specialist care unit(s) normally referred to were closed for a period of time<br>f. People were reluctant to be linked<br>g. Other (please specify):		
In the table below, please provide the percentage of persons testing/screening positive in your site who were linked to care in the indicated time periods<br>Please provide your best estimate or percentage range, but enter as a percentage. If no data is available, please type in 'No data'			
	March 2019 (baseline)	March-May 2020	June-August 2020
HIV			
HBV			
HCV			

Syphilis			
Chlamydia			
Gonorrhoea			

(NOT FOR NATIONAL LEVEL RESPONDENTS OR LABORATORIES)

Impact of COVID-19 on the continuum of care

In comparison with the same three months last year (1 March to 31 May 2019), In the table below, please indicate the relative change in the following components of the continuum of care in your service/site in the period 1 March to 31 May 2020 (if accurate data are not available then please provide your best estimate/judgment):

	Decreased by			Stable	Increased by			Not applicable for my facility/site	No data
	>50%	26-50%	11-25%	0-10% (+/-)	11-25%	26-50%	>50%		
Time from blood draw to receipt of confirmatory test result from the laboratory (diagnostic tests)									
Time from blood draw to receipt of clinical monitoring test results from the laboratory (e.g. viral load, liver enzymes, CD4 etc.)									
Time from confirmatory diagnosis to treatment initiation<br>• For HIV<br>• For HCV									
Please elaborate on your response as relevant				*Free text field*					

Impact of COVID-19 on the continuum of care

In comparison with the same three months last year (1 June to 31 August 2019), In the table below, please indicate the relative change in the following components of the continuum of care in your service/site in the period 1 June to 31 August 2020 (if accurate data are not available then please provide your best estimate/judgment):

	Decreased by	Stable	Increased by	Not applicable for my facility/site	No data

	>50%	26-50%	11-25%	0-10% (+/-)	11-25%	26-50%	>50%		
Time from blood draw to receipt of confirmatory test result from the laboratory (diagnostic tests)									
Time from blood draw to receipt of clinical monitoring test results from the laboratory (e.g. viral load, liver enzymes, CD4 etc.)									
Time from confirmatory diagnosis to treatment initiation<br>• For HIV<br>• For HCV									
Please elaborate on your response as relevant				*Free text field*					

(ONLY FOR RESPONDENTS WHO INDICATED PREP SERVICES)
Impact on HIV pre-exposure prophylaxis (PrEP) provision
In comparison with the same time period last year (period 1 March to 31 August 2019), please indicate the relative change in the number of persons on PrEP in your service/site in the period 1 March to 31 August 2020 (if accurate data are not available then please provide your best estimate):

	Decreased by			Stable	Increased by			No data
	>50%	26-50%	11-25%	0%-10% (+/-)	11-25%	26-50%	>50%	
Number of new persons initiating PrEP								
Total number of persons on PrEP								
Please elaborate on your response as relevant				*Free text field*				

**Changes or adaptations put in place to mitigate the impact of COVID-19 on testing for HIV, viral hepatitis and STIs in your facility/site**	
(ONLY FOR LABORATORIES)<br>Please briefly describe what measures were put in place to mitigate the impact of COVID-19 on the provision of testing for HIV, HCV, HBV and STIs in your laboratory?	*Please describe (free text field)*

(NOT FOR NATIONAL LEVEL RESPONDENTS OR LABORATORIES)<br>**New measures implemented to restore testing provision in response to COVID-19**	
What measures were put in place to restore the provision of (diagnostic) testing services for HIV, viral hepatitis and STIs in your facility/setting <u>in response to the COVID-19 pandemic</u> (please check all that apply)?	a. Remote counselling appointments via phone or online<br>b. Home-based sampling (if yes, please indicate for which infections)<br>c. HIV self-testing (offered on-site or referred to other service – online/pharmacy)<br>d. Triaging of patients (stricter criteria for who is being offered testing)<br>e. No 'drop-in' service (only testing by appointment)<br>f. Referral to other sites if testing could not be performed at your facility<br>g. Staff reinforcement<br>h. Funding reallocations<br>i. Equipment acquisition (purchasing of new testing platforms)<br>j. Expanded outreach testing<br>k. Testing campaigns<br>l. Other (please specify)
If NOT currently offered at your site, please indicate if you are planning to introduce any of the measures listed in the coming year (please indicate which)	a. Remote counselling appointments via phone or online<br>b. Home-based sampling (if yes, please indicate for which infections)<br>c. HIV self-testing (offered on-site or referred to other service – online/pharmacy)<br>d. Triaging of patients (stricter criteria for who is being offered testing)<br>e. No 'drop-in' service (only testing by appointment)<br>f. Referral to other sites if testing could not be performed at your facility<br>g. Staff reinforcement<br>h. Funding reallocations<br>i. Equipment acquisition (purchasing of new testing platforms)<br>j. Expanded outreach testing<br>k. Testing campaigns<br>l. Other (please specify)
1. If HIV self-testing is being offered at your facility/site, please indicate how it is implemented (please tick all that apply)	a. On-site distribution of self-testing kits<br>b. Self-testing is provided by referral but kits need to be purchased elsewhere (pharmacy, online etc.)<br>c. We offer counselling and referral to confirmatory testing/linkage to care for people with a positive self-test result<br>d. Do now know<br>e. Other (please specify)
Please elaborate on your response as relevant	*Free text field*

(NOT FOR NATIONAL LEVEL RESPONDENTS OR LABORATORIES)

Changes in testing or counseling performed remotely, through self-testing or home-based sampling

Please indicate what percentage of people being counselled for HIV, viral hepatitis or STI (diagnostic) testing, who were offered the following in the indicated time periods.

Please provide your best estimate or percentage range, but enter as a percentage. If no data is available, please type in 'No data'

	March 2019 (baseline)	March-May 2020	June-August 2020
Percentage of clients receiving testing counselling who received it remotely (via phone or online)?			
Percentage of clients receiving testing counselling who were offered or referred to HIV self-testing?			
Percentage of clients receiving testing counselling who were offered home-based sampling?			
Please elaborate on your response as relevant	*Free text field*		

(ONLY FOR RESPONDENTS WHO INDICATED PREP SERVICES)<br>**New measures implemented to support continued provision of PrEP**	
If you provide follow-up for people on PrEP, did you implement any of the following changes in PrEP provision after the pandemic declaration?	a. Reduced frequency of follow-up visits<br>b. Reduced frequency of clinical monitoring tests (viral load, liver enzymes etc.)<br>c. Reduced number of clinical tests performed on each patient sample (to avoid overburdening laboratories)<br>d. Possibility of home-based sampling<br>e. Telemedicine (phone/online consultations or e-prescriptions)<br>f. Multi-month prescriptions/increased quantity of medicine dispensed per visit<br>g. Additional locations for pill pickup (if yes, please specify)<br>h. Home delivery<br>i. Other
If you provided "Additional locations for pill pickup," please specify	*Free text field*

**Specific questions for the primary care sector**	
How was your work affected by the COVID-19 pandemic during the period 1 March to 31 May 2020 (tick all that apply)	a. Only patients with acute health problems could attend<br>b. No referral to specialists was possible, except emergency situations<br>c. No elective procedures/ investigations were possible, except emergency situations<br>d. Other
If other, (please specify other impacts on your ability to provide testing and care for HIV, viral hepatitis and STIs).	
Is HIV/HBV/HCV/STI testing recommended at the primary health care level in your country?	a. Yes<br>b. No
If yes, please specify testing recommendations before and after the COVID-19 pandemic:	

	Pre-COVID testing recommendations	Post-COVID testing recommendations	Do not know
Routinely offered to all			
Offered to people presenting with an indicator condition			
Offered to key populations at higher risk of infection			
Offered on demand			
Other			

**Changes or adaptations put in place to mitigate the impact of COVID-19 on testing and care for HIV, viral hepatitis and STIs in your facility/site**	
After the pandemic declaration, did you implement any of the following changes to the clinical care/follow-up and disease monitoring practice <u>for your HIV/HBV/HCV/STI patients</u>?	a. Reduced frequency of follow-up visits<br>b. Reduced frequency of clinical monitoring tests (viral load, liver enzymes etc.)<br>c. Reduced number of clinical tests performed on each patient sample (to reduce workload on laboratories)<br>d. Possibility of home-based sampling<br>e. Telemedicine (phone/online consultations or e-prescriptions)<br>f. Multi-month prescriptions/increased quantity of medicine dispensed per visit<br>g. Additional locations for pill pickup (if yes, please specify)<br>h. Home delivery (of any medicine)<br>i. Other
Please elaborate on your response as relevant	*Free text field*

**Specific questions for the Secondary Care level/Specialty settings**	
Does your specialty recommend (diagnostic) testing for HIV, viral hepatitis and STIs in your setting?	a. Yes<br>b. No
If yes, please specify testing recommendations before and after the COVID-19 pandemic:	

	Pre-COVID testing recommendations	Post-COVID testing recommendations	Do not know
Routinely offered to all			
Offered to people presenting with an indicator condition			
Offered to key populations at higher risk of infection			
Offered on demand			
Other			
Please use this field to elaborate on your response as relevant			

**Impact of COVID-19 in Secondary Care level/Specialty settings**<br><br>Please indicate the percentage of patients who had a clinic appointment who did not show up or were not reachable by phone or other means in your facility/site in the indicated time periods listed below:<br>Please provide your best estimate or percentage range, but enter as a percentage. If no data is available, please type in 'No data'	
March 2019 (Baseline)	
March - May 2020	
June - August 2020	

Please elaborate on your responses as relevant	
After the pandemic declaration, did you implement any of the following changes to the clinical care/follow-up and disease monitoring practice for your HIV/HBV/HCV/STI patients?	a. Reduced frequency of follow-up visits<br>b. Reduced frequency of clinical monitoring tests (viral load, liver enzymes etc.)<br>c. Reduced number of clinical tests performed on each patient sample (to reduce workload on laboratories)<br>d. Possibility of home-based sampling<br>e. Telemedicine (phone/online consultations or e-prescriptions)<br>f. Multi-month prescriptions/increased quantity of medicine dispensed per visit<br>g. Additional locations for pill pickup<br>h. Home delivery (of any medicine)<br>i. Other
If "Yes" to "Additional locations for pill pickup," please specify	
Please elaborate on your response as relevant	

**Specific questions for Community-based organisations/sites**	
What are the main key groups/communities that access your services? (Select all that apply):	a. Young people<br>b. General population<br>c. Gay, bisexual and other men having sex with men<br>d. Transgender people<br>e. Sex workers<br>f. People who inject drugs<br>g. Migrants<br>h. People living with HIV<br>i. Other (please specify)
Did your clients report increased needs and/or any new needs since the pandemic declaration?	a. Yes<br>b. No
If "Yes," please indicate which increased and/or new needs that have been expressed? (Select all that apply)	a. Financial support<br>b. Social support<br>c. Mental health support<br>d. Housing support<br>e. Transportation<br>f. Food insecurity<br>g. Difficulty accessing health services<br>h. Other (Please specify)

In comparison with the period 1 March to 31 May 2019, please indicate the relative change in the volume of services provided by your site in the period 1 March to 31 August 2020 (please provide your best estimate):

	Decreased			Stable	Increased			No data
	>50 %	26-50%	11-25%	0%-10% variation (up or down)	11-25%	26-50%	>50 %	
Mental health support								
Social support								
Opioid substitution therapy (OST)								

Needle and syringe exchange								
Partner notification								

Service adaptations

In comparison with the period **1 March to 31 May 2019**, please indicate if you changed the way you provided these services in the period **1 March to 31 August 2020**:

	No, we offer it the same as before	We do it online now	We do it over the phone now	We do it by appointment only now	We refer it to other organizations
Mental health support					
Social support					
Opioid substitution therapy (OST)					
Needle and syringe exchange					
Partner notification					

**Economic effects**	
Is your organization suffering from budget cuts due to the COVID-19 epidemic?	a. Yes<br>b. No
If yes, what is the approximate % value of your annual budget that was cut?	a. over 75%<br>b. 50-75%<br>c. 26-50%<br>d. 11-25%<br>e. 0-10%
**COVID-19 testing**	
Have you considered adding COVID-19 testing to your services?	a. We haven't thought about it<br>b. We don't think it makes sense for us<br>c. We do not think it will be possible<br>d. Yes, we would be willing to incorporate the offer of COVID-19 rapid tests<br>e. We are already performing COVID-19 testing<br>f. Other (Please specify)
If "Yes," what kind of tests would you be willing to incorporate (or are you already using)?	a. Serological rapid tests<br>b. Antigen rapid tests<br>c. Both types of rapid tests<br>d. Sample collection for PCR<br>e. Other (Please specify)
Why do you think it is not going to be possible to incorporate COVID-19 testing? (Select all that apply)	a. We do not have resources to buy the tests<br>b. We are not going to be able to get the tests<br>c. Our service is not going to be allowed to perform<br>d. the tests<br>e. Only health care professionals are allowed to<br>f. perform the tests<br>g. Other (Please specify)

**Main negative and positive consequences, service adaptations and lessons learned**	
In your opinion, what were the major **negative** consequences of the COVID-19 pandemic on your provision of HIV, viral hepatitis and STIs prevention, testing and care services?	*Please describe (free text field)*
In your opinion, what were the main **positive** consequences of the COVID-19 pandemic on your provision of HIV, viral hepatitis and STIs prevention, testing and care services and will any of the positive lessons learned continue to impact how services will be provided in the future?	*Please describe (free text field)*
(ONLY FOR NATIONAL LEVEL RESPONDENTS)<br>In your opinion, what were the major negative consequences of the COVID-19 pandemic on your country's HIV, viral hepatitis and STIs prevention, testing and care services?	*Please describe (free text field)*
(ONLY FOR NATIONAL LEVEL RESPONDENTS)<br>In your opinion, what were the main positive consequences of the COVID-19 pandemic and will service adaptations/positive lessons learned continue to impact how services will be provided in the future?	*Please describe (free text field)*

(ONLY FOR NATIONAL LEVEL RESPONDENTS)<br>**Financial impact**	
Were there financial cuts in the public funding to the HIV/Hep/STI response as a result of the COVID pandemic?	a. Yes<br>b. No<br>c. No data available<br>d. Do not know

**Support needs going forward**	
Is there any specific guidance or support that you would consider important in the coming months to reduce the impact of COVID-19 on testing for HIV, viral hepatitis and STIs in your facility/setting? (select all that apply)	a. Additional human resources<br>b. Increased financial support<br>c. Regulatory changes (please elaborate)<br>d. Programmatic guidance (free text)<br>e. Technical guidance (free text)<br>f. Technical support on specific issue (free text)<br>g. Procurement/supply chain related support (free text for details)<br>h. Other (specify which other – free text)<br>i. No, none
Please elaborate on your response as relevant	*Free text field*
Please use this space to provide any additional comments, links to resources, etc.	

Supplementary Table S2. COVID-19 impact assessment on testing for HIV, viral hepatitis and STIs in the WHO European Region: Distribution of respondents by country and type (n=98)

Country	Setting (respondent type)			Total
	Secondary level/specialist care	Community-based organisation	National public health institute or Ministry of Health	
Albania	0	1	0	1
Armenia	0	1	0	1
Austria	1	0	0	1
Belgium	0	5	0	5
Bosnia and Herzegovina	1	0	0	1
Croatia	0	1	0	1
Cyprus	0	1	0	1
Denmark	1	1	1	3
Estonia	1	1	0	2
France	0	0	1	1
Georgia	1	2	1	4
Germany	3	5	1	9
Greece	0	1	0	1
Hungary	1	0	0	1
Iceland	1	0	0	1
Ireland	3	0	1	4
Israel	2	1	0	3
Italy	3	3	0	6
Lithuania	2	1	0	3
Malta	1	0	0	1
Montenegro	0	0	1	1
Netherlands	1	0	1	2
Norway	1	1	0	2
Poland	2	1	1	4
Portugal	0	2	0	2
Romania	2	1	0	3
Russia	1	0	0	1
Serbia	0	1	1	2
Slovakia	0	2	0	2
Slovenia	0	1	0	1
Spain	1	10	1	12
Switzerland	1	1	0	2
Ukraine	2	5	0	7
United Kingdom	4	3	0	7
**Total**	**36**	**52**	**10**	**98**
